# Single cell phototransfection of mRNAs encoding SARS-CoV2 spike and nucleocapsid into human astrocytes results in RNA dependent translation interference

**DOI:** 10.3389/fddev.2024.1359700

**Published:** 2024-03-05

**Authors:** Hyun-Bum Kim, Quentin Brosseau, Julia Radzio, Jinhui Wang, Hiromi Muramatsu, Da Kuang, M. Sean Grady, H. Isaac Chen, John A. Wolf, Alexandra V. Ulyanova, Tamas Bartfai, Junhyong Kim, Norbert Pardi, Jai-Yoon Sul, Paulo Arratia, James Eberwine

**Affiliations:** ^1^ Department of Systems Pharmacology and Translational Therapeutics, University of Pennsylvania, Philadelphia, PA, United States; ^2^ Department of Mechanical Engineering and Applied Mechanics, University of Pennsylvania, Philadelphia, PA, United States; ^3^ Department of Microbiology, Perelman School of Medicine, University of Pennsylvania, Philadelphia, PA, United States; ^4^ Department of Biology, University of Pennsylvania, Philadelphia, PA, United States; ^5^ Department of Neurosurgery, Perelman School of Medicine, University of Pennsylvania, Philadelphia, PA, United States

**Keywords:** mRNA translation, SARS-CoV-2, transcriptome induced phenotype remodeling, phototransfection, transltion competition

## Abstract

Multi-RNA co-transfection is starting to be employed to stimulate immune responses to SARS-CoV-2 viral infection. While there are good reasons to utilize such an approach, there is little background on whether there are synergistic RNA-dependent cellular effects. To address this issue, we use transcriptome-induced phenotype remodeling (TIPeR) via phototransfection to assess whether mRNAs encoding the Spike and Nucleocapsid proteins of SARS-CoV-2 virus into single human astrocytes (an endogenous human cell host for the virus) and mouse 3T3 cells (often used in high-throughput therapeutic screens) synergistically impact host cell biologies. An RNA concentration-dependent expression was observed where an increase of RNA by less than 2-fold results in reduced expression of each individual RNAs. Further, a dominant inhibitory effect of Nucleocapsid RNA upon Spike RNA translation was detected that is distinct from codon-mediated epistasis. Knowledge of the cellular consequences of multi-RNA transfection will aid in selecting RNA concentrations that will maximize antigen presentation on host cell surface with the goal of eliciting a robust immune response. Further, application of this single cell stoichiometrically tunable RNA functional genomics approach to the study of SARS-CoV-2 biology promises to provide details of the cellular sequalae that arise upon infection in anticipation of providing novel targets for inhibition of viral replication and propagation for therapeutic intervention.

## Introduction

The use of mRNA as a modulator of cell biology is still in its infancy (Weng et al., 2020; Qin et al., 2022; Qureischi et al., 2022; Khorkova et al., 2023), with some examples including its use in cell phenotype engineering (Sul et al., 2009; Angel and Yanik, 2010; Kim et al., 2011; Diener et al., 2015; Billingsley et al., 2020; Moradian et al., 2020), to add back or increase protein functionality (Robinson et al., 2018; Miao et al., 2020) and as a means to produce antigens to elicit an immune response (Kallen and Theß, 2014; Pardi and Weissman, 2017; Pardi et al., 2018; Verbeke et al., 2019; Freyn et al., 2020; Zhang et al., 2021; Pardi et al., 2022). Among the advantages of using mRNA to study biological processes is that it is transient in its expression and will disappear from cells using a cells natural degradation process and there is no need for it to enter the nucleus to be transcribed as is the case for DNA mediated therapeutics (Kulkarni et al., 2021). Disadvantages include that it is more labile than DNA and hence is challenging to experimentally manipulate and to introduce into cells due to its highly charged backbone. Focus on the use of mRNA as a therapeutic modality has intensified because of the success of the SARS-CoV-2 mRNA vaccine (discussed below).

Notably, the body mounts an immune response when the presence of an antigen is detected. The antigen may be presented by infection with the natural pathogen, exogenously administered pathogen protein/peptide fragment or translation of the antigen-encoding mRNA provided by mRNA or DNA infection or transfection. The direct mRNA transfection approach has been used over the last 2 decades (Leitner et al., 1999; Pascolo, 2004; Pardi et al., 2018; Zhang et al., 2021), most notable recently in the generation of the SARS-CoV-2 mRNA vaccines (Baden et al., 2020; Li et al., 2020; Thanh Le et al., 2020; Walsh et al., 2020; Turner et al., 2021; Hogan and Pardi, 2022). In this case, the mRNA that was transfected into host cells encoded the Spike glycoprotein of SARS-CoV-2, which when expressed from the mRNA induced cellular and humoral immune responses (Grifoni et al., 2020; Hogan and Pardi, 2022). Most mRNA vaccines rely upon a single mRNA encoding the antigen of interest. While the single antigen SARS-CoV-2 vaccine has been highly successful in stimulating an immune response, it may not be sufficient to induce broadly protective responses against certain pathogens (Pascolo, 2004). As shown in decades of immunological work, many of the most successful vaccines derive from the use of multiple antigenic sites from the foreign entity (Burrell et al., 2017). By analogy with this previous work and as anticipated by several studies (Freyn et al., 2020; Sajid et al., 2021; Arevalo et al., 2022; McMahon et al., 2022; Pardi et al., 2022) it is expected that co-transfection of multiple mRNAs encoding different antigens of a pathogen may elicit a synergistic protective immune response.

The potential for multi-RNA transfection to induce protective immunity has been explored for the SARS-CoV-2 virus through the creation of bivalent mRNA vaccines with each RNA encoding different versions of the Spike proteins. Among the proteins encoded by in the SARS-CoV-2 ∼30 kb sense strand RNA (Xia et al., 2020; Hu et al., 2021) are the Spike protein, which is a viral surface protein involved in viral attachment and entry of the virus into the cell, and the Nucleocapsid protein (Nuc) which is involved in RNA packaging and is in the viral capsid. Both of these RNAs have been introduced simultaneously into cells (Dangi et al., 2021; Brouwer et al., 2021) via lipid nanoparticle (LNP) transfection (Reichmuth et al., 2016; Brouwer et al., 2021; Schoenmaker et al., 2021). Indeed the use of these two RNAs produced an enhanced immune response, including higher titer antibodies observed in mice (Dangi et al., 2021). This multi-viral protein encoding RNA strategy has also been explored to create a vaccine against human immunodeficiency virus (HIV) where the Env and Gag-encoding mRNAs were used to generate HIV-1 neutralizing antibodies (Zhang et al., 2021).

The expression of multiple proteins may also provide a means to better understand the biological consequences of expression of the viral proteins upon infection. These proteins may interact with each other or cellular proteins or RNAs, altering the biology of the host cell. To assess cellular consequences to infection, the experimental proteins need to be co-expressed in the same cell in such a manner that the protein abundances can be manipulated to enhance antigen presentation. As protein translation often correlates with its mRNA abundance, the easiest way of modulating the abundances of desired proteins is to vary the quantity of mRNAs encoding the protein of interest. In the case of viral infection, the impact of expression of the mRNAs encoded by the viral genome can be assessed by introducing these mRNAs into the recipient cell in user-defined abundances. Co-expression of multiple exogenous RNAs in individual cells has been performed previously but with little concern about the relative levels of RNA transfection and subsequent protein expression (Class et al., 2021; Brouwer et al., 2021). For example, addition of multiple mRNAs has been used to induce the conversion of differentiated cells into induced pluripotent stem cells. The efficiency of this process is low likely due to inefficient introduction of the mRNAs into cells and/or because specified ratios of abundances of mRNAs/proteins necessary to produce the drivers of differentiation were not recapitulated upon transfection. Alternatively, addition of an isolated transcriptome from a particular cell type can be transfected into a host cell and the recipient cell’s phenotype will change to that of the mRNA donor cell (Barrett et al., 2006; Kim J. and Eberwine, 2010; Kim et al., 2011). These studies show that a cell’s transcriptome contains the information (phenotypic memory) that can confer cell phenotype upon the cell.

Analysis of the regulatory constraints on co-translation of multiple mRNAs requires that specified amounts of mRNA be introduced into cells. Chemical, viral and physical transfection methods have been used to transfect cells with each having its advantages (Kim T. K. and Eberwine, 2010). The recent success of the COVID-19 vaccine lies in part with the use of LNP that encapsulate mRNA and facilitate its cellular uptake while providing adjuvant activity (Pardi et al., 2015; Oberli et al., 2017; Alameh et al., 2021). While successful, if the goal is to introduce differing abundances of mRNAs into cells, nanoparticles are not ideal as the particles which can vary in size have the same ratio of a single type of mRNA to particle size. Further, there are reports of LNP carriers in preclinical and clinical studies causing side effects including being pro-inflammatory responses (Moghimi, 2021; Guerrini et al., 2022) which may be important for eliciting immunity but can be problematic for non-immune related biologies. Other physical methods that do not utilize carriers often offer fewer side-effects and are more amenable to introducing differing types and amounts of RNAs, but have their own sets of issues (Kim T. K. and Eberwine, 2010). One such method, laser-mediated transfection (also known as phototransfection) uses a pulse laser to irradiate a cell membrane to form a transient pore (Barrett et al., 2006). Nucleic acids present in the medium diffuse through the pores into the cell due to the osmotic difference between the culture medium and the cellular cytosol. The optical method enables observation of the recipient cell that is to be transfected and to make pores at any location on the recipient cell. In contrast to DNA-mediated transfection where mRNA expression is controlled by promoter activation, it is possible to adjust protein expression levels simply by changing the amount of mRNA transfected and the frequency of phototransfection (Barrett et al., 2006; Kim J. and Eberwine, 2010; Kim et al., 2011). Other advantages of mRNA transfection include: 1) transfected mRNAs can be expressed within minutes after transfection skipping translocation of a cDNA containing plasmid into, and transcription in the nucleus and 2) transcriptomes (population of mRNAs) can be transfected into and expressed in host cells, which is practically difficult for DNA transfection. However, while laser-based transfection can be envisioned as a preclinical approach to understanding aspects of RNAs impact upon cell biology or as a personalized therapeutic approach to treat certain diseases or tissues, it is untenable to immunize hundreds of millions of people in a pandemic scenario as has done with LNPs.

Herein, we address the hypothesis that two mRNAs from the SARS-CoV-2 virus can synergistically interact in a concentration dependent manner to effect host cell viral protein expression. We show that individual human, mouse, and rat cells can be phototransfected with multiple mRNAs whereupon they are translated into protein in an mRNA concentration-dependent manner. Further, the abundances of individual transfected mRNAs can impact the number of translated proteins from other co-transfected mRNAs. TIPeR-mediated expression of varying but known, concentrations of multiple SARS-CoV-2 mRNAs in single human, which have been shown to be infected SARS-CoV-2 (Crunfli et al., 2021; Andrews et al., 2022) provides a unique means to better modulate antigen presentation in host cells and, in expanded studies, to understand the cellular mechanisms by which coronavirus’s elicit specific and distinct cellular consequences. This provides a rationale for high-throughput analysis to screen for effects of systematically varying the abundance of members of cohorts of mRNAs to assess the systems biology of single cell RNA landscapes.

## Results

**FIGURE 1:**
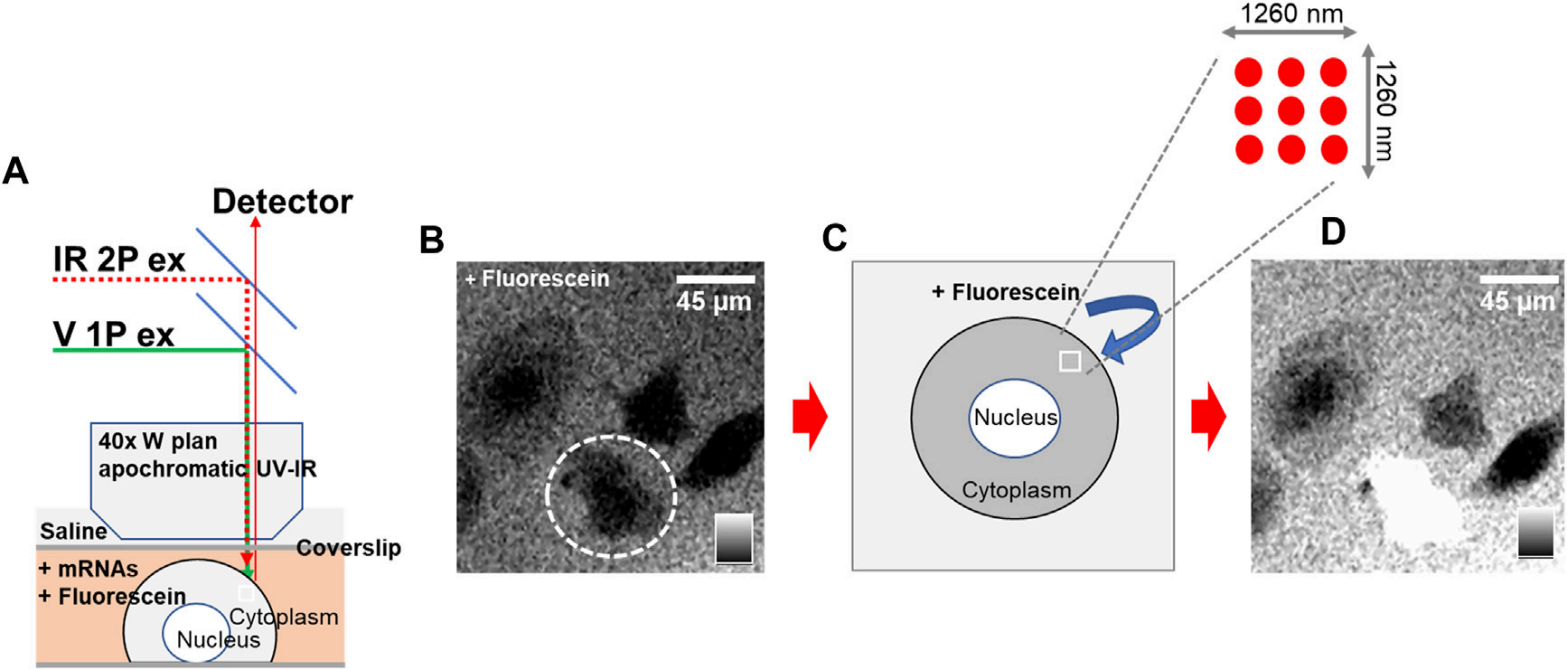
Phototransfection Process and Visualization in Rat PC12 Cells – **(A)** Schematic representation of the phototransfection set-up. **(B)** An intact PC12 cell is circled and appears black entity (lacking fluorescence) as it is surrounded by fluorescein which is excluded from the cell. **(C)** Schematic illustration demonstrating the phototransfection irradiation process using multiple laser light pulses in a 3 × 3 pattern over 1,260 nm^2^ of the cell membrane. This process introduces transient holes into the cell membrane. **(D)** Following laser irradiation, fluorescein diffuses into the cell through the holes created in the cell membrane. The PC12 cell now appears white due to fluorescence of the internalized fluorescein. Scale bar is 45 microns.

Phototransfection utilizes laser light to transiently introduce holes into a cell allowing extracellular material such as mRNA to move into the cell (Barrett et al., 2006; Sul et al., 2009). The phototransfection process requires imaging of the host cell so that the infrared laser can be focused on the surface of the cell membrane to limit the amount of photon induced damage to the cell, schematized in Figure 1A. We confirmed the laser settings are appropriate for phototransfection of cells by checking for dye introduction into cells and survival of cells. Among the several phototransfection parameters that are optimized to enhance transfection while limiting adverse cell effects is the distance of the phototransfecting laser point source from the host cell membrane. Data in Supplementary Figure S1 shows that sub-optimal focusing can result in reduced transfection efficiency or even cell death. Such optimization experiments are conducted at the beginning of every experiment to identify the most appropriate experimental parameters (Sul et al., 2009; Raes et al., 2020). The phototransfection process is visualized in control experiments where membrane-impermeable fluorescein is introduced into a rat PC12 cell line (Figures 1B–D). In Figure 1B, cultured PC12 cells are bathed in fluorescein and appear as black entities (no fluorescence). Upon irradiation with multiple pulses of laser light in a 3 × 3 pattern over 1,260 nm^2^ (schematized in Figure 1C), fluorescein diffuses through the light induced holes in the cell membrane and the cell appears as white because of the fluorescein that diffused into the cell. Note that other cells that were not laser-irradiated remain nonfluorescent.

**FIGURE 2:**
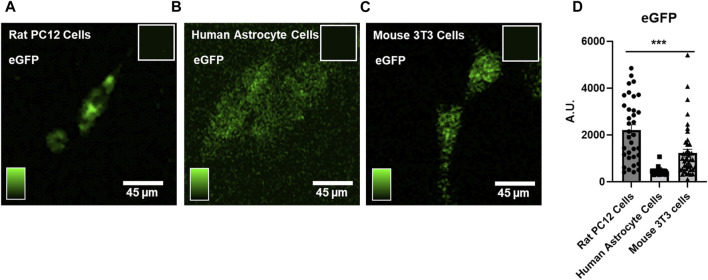
Phototransfection of eGFP mRNA into Rat PC12 Cells, Human Astrocytes and Mouse 3T3 cells – **(A)** A phototransfected PC12 cell appears green from expression of eGFP protein after phototransfection with eGFP-encoding mRNA (0.38 μg/mL in the PBS surrounding the cell). Inset shows lack of fluorescence in nontransfected PC12 cells. **(B)**Two human astrocytes exhibit eGFP protein fluorescence after phototransfection with eGFP mRNA (0.38 μg/mL). Inset shows lack of fluorescence signal in nontransfected human astrocyte cells. **(C)** Mouse 3T3 cells also express eGFP after phototransfection (0.38 μg/mL). **(D)** Quantification of eGFP fluorescence data from multiple cell phototransfections. PC12 cells (N = 39) exhibited higher eGFP expression compared to human astrocytes (N = 59) and mouse 3T3 cells (N = 43). Data are presented as mean ± SEM. ***, *p* < 0.001. Scale bar is 45 microns.

Phototransfection allows essentially any molecule including mRNA to diffuse the cell that is laser-irradiated as evidenced by the observed eGFP protein fluorescence in the rat PC12 cell line (Figure 2A), human astrocytes in primary cell culture (Figure 2B), and mouse 3T3 cells (Figure 2C) after phototransfection of eGFP-encoding mRNA. All cell types were phototransfected with 0.38 μg/mL mRNA. The more higher GFP fluorescence in PC12 cells suggests that there is differential translational efficacy for eGFP between the three species, with the lowest efficiency observed in human astrocytes (Figure 2D). These data highlight that cell species is an important factor in assessing the RNA functional biology potentially due to differences in endogenous cell transcriptome landscape.

**FIGURE 3:**
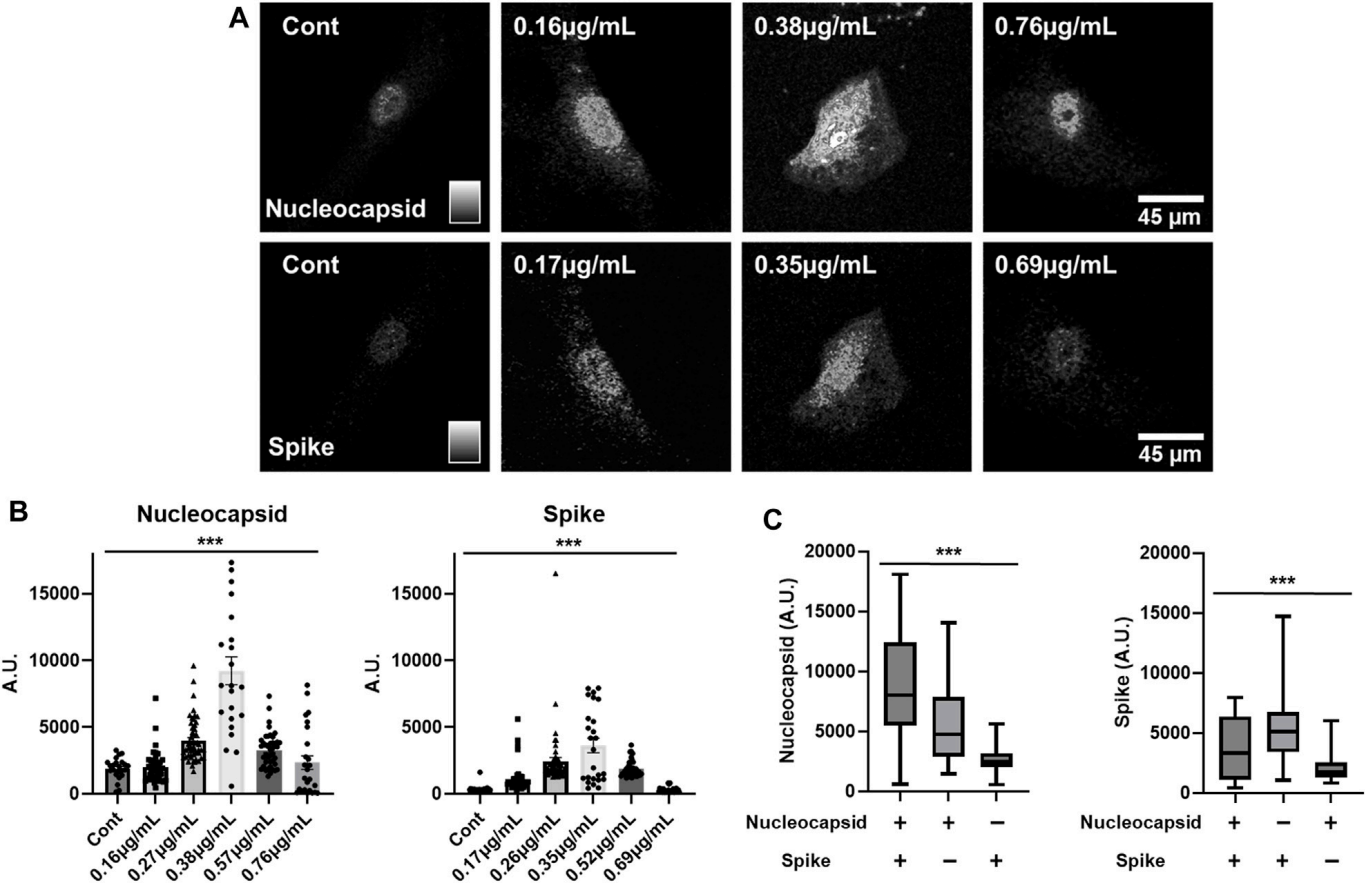
mRNA Concentration Dependence of Translation of SARS-CoV-2 Spike and Nuc mRNAs in Human Astrocytes **– (A)** Immunostaining of control and phototransfected primary cultures of human astrocytes that were simultaneously transfected with Nuc and Spike mRNAs. Both viral proteins display nuclear and cytoplasmic localization. Scale bar is 45 microns. **(B)** Concentration-dependent expression of phototransfected Nuc and Spike mRNAs. From left-to-right the bars show increasing RNA concentrations (Nuc mRNA: Cont. N = 35, 0.16 μg/ml. N = 59, 0.27 μg/ml. N = 67, 0.38 μg/ml. N = 35, 0.57 μg/ml. N = 52, 0.76 μg/ml. N = 35. Spike mRNA: Cont. N = 35, 0.17 μg/ml. N = 59, 0.26 μg/ml. N = 67, 0.35 μg/ml. N = 35, 0.52 μg/ml. N = 52, 0.69 μg/ml. N = 35). **(C)** The influence of co-expression of COVID-19 Nuc and Spike mRNAs on protein expression is shown. Left-most graph shows increased Nuc protein expression when Nuc mRNA and Spike RNA were phototransfected. The right-most graph shows Spike protein expression when only Spike RNA was phototransfected. Co-phototransfection also reduces Spike protein expression (Nuc mRNA (+), 0.38 μg/mL/Spike mRNA (+), 0.35 μg/ml N = 35, Nuc mRNA (+), 0.38 μg/mL/Spike mRNA (−), 0 μg/ml N = 70, Nuc mRNA (−), 0 μg/mL/Spike mRNA (+), 0.35 μg/ml N = 70). The *Y*-axis is arbitrary units. Data are presented as mean ± SEM. ***, *p* < 0.001.

SARS-CoV-2 infects several human cell types through association with the cell surface angiotensin converting enzyme 2 (ACE2) (Zamorano Cuervo and Grandvaux, 2020; Shirbhate et al., 2021), but in the central nervous system preferentially transfects astrocytes over neurons (Crunfli et al., 2021; Song et al., 2021; Andrews et al., 2022; Huang and Fishell, 2022). To better understand the process of COVID-19 infection in humans, we sought to assess the dynamics of translation of different SARS-CoV-2 protein-encoding mRNAs in human cells. To this end, human astrocytes were used as host cells to assess the translation of phototransfected SARS-CoV-2 mRNAs encoding the Nucleocapsid (Nuc) and Spike proteins. The astrocytes were from human cortical primary cell cultures derived from neurosurgical resections (Spaethling et al., 2017) from patients with epilepsy and recurrent seizures. After 7 weeks in culture, the astrocytes were removed and replated in gridded chambers for 2 days, at which point they were phototransfected. Three days after phototransfection, the astrocytes were fixed and immunostained with antibodies to the Nuc and Spike proteins. The immunostaining of control and cells that had been simultaneously phototransfected with both mRNAs shows expression of both the Nuc and Spike proteins (Figure 3A). Nuclear and cytoplasmic localization of each of the viral proteins is apparent similar to what has been reported previously for proteins made after LNP-mediated Spike and Nuc mRNA transfection in other cell types (Wurm et al., 2001; Zhang et al., 2021; Chen et al., 2022; Sattar et al., 2023). These data show that human astrocytes in primary cell culture can be phototransfected and that the introduced SARS-CoV-2 protein-encoding mRNAs can be translated.

The expression of transfected mRNAs is concentration-dependent (Figure 3A) with increasing concentrations from the left most panel to the right most with the peak expression for both Nuc and Spike mRNAs being 0.38 and 0.35 μg/mL respectively (Figure 3B). At higher concentrations (Figure 3A, right panels), there was significantly less expression for both mRNAs (Figure 3B). These data suggest that high concentrations of mRNA inhibit the translation of the transfected mRNA.

We next explored how the co-expression of these SARS-CoV-2 protein-encoding mRNAs might influence the expression of each other. Figure 3C shows the expression of Spike protein when only Spike mRNA is phototransfected as well as Nuc protein when only Nuc mRNA is introduced into the cells. When phototransfected individually, each of these mRNAs yield approximately the same level of detectable protein. When these mRNAs are co-phototransfected with the same amount of mRNA as used in the single mRNA transfections, the expression of Spike protein is reduced (∼0.5 fold) while the amount of Nuc protein is increased (∼1.5 fold). These data suggest that translation of Nuc protein inhibits the translation of Spike protein while Spike mRNA translation enhances Nuc translation creating an infection-associated cellular environment that may be important for SARS-CoV-2 expression and infection.

**FIGURE 4:**
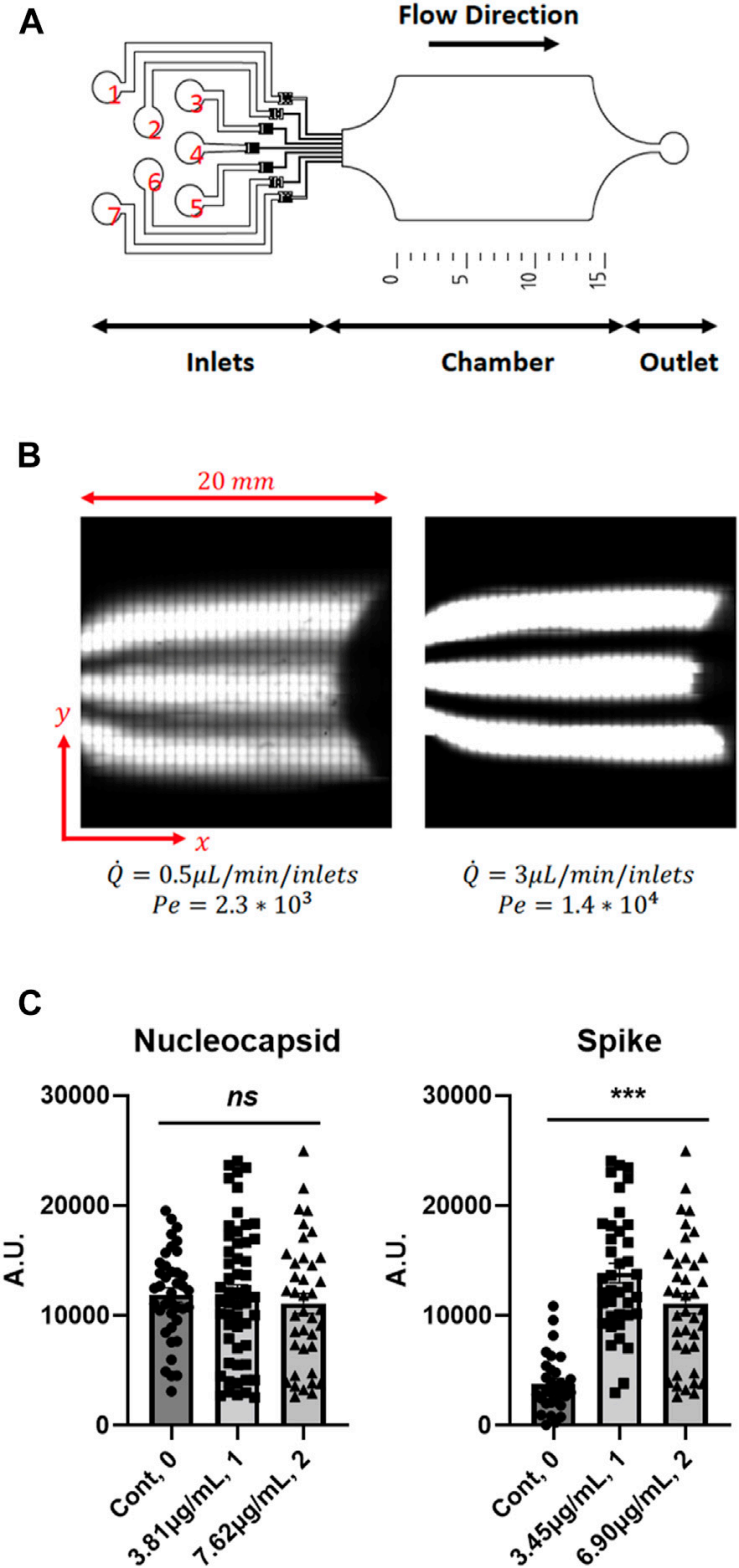
Microfluidics Chamber Phototransfection of Multiple RNAs into Mouse 3T3 Cells - **(A)** Schematic of the high-throughput microfluidic device with 7 inlets, chamber, and 1 outlet. **(B)** Example of laminar flow in the chamber of the microfluidic device chamber. Diffusion and mixing of a fluorophore perpendicular to the flow direction depends on the Péclet number. **(C)** Concentration-dependent expression of transfected RNAs. (Nuc mRNA: Cont. N = 39, 3.81 μg/ml. N = 54, 7.62 μg/ml. N = 39. Spike mRNA: Cont. N = 31, 3.45 μg/ml. N = 39, 6.90 μg/ml. N = 39). Data are presented as mean ± SEM. *ns*, not significant. ***, *p* < 0.001.

As these data show that phototransfected mRNA is translated in cells in a concentration dependent manner, we sought to develop a high-throughput microfluidic device to systematically modify the abundances of phototransfected RNAs to assess the mRNA concentration dependences of cells. The high-throughput, two layer, reversibly sealable microfluidic device used in this study consists of seven inlets, a shallow-flow chamber for cell visualization and phototransfection, and an outlet (Figure 4A). The microfluidic device operating parameters are the Reynolds (*Re*) and Peclet (*Pe*) numbers. The Reynolds number is defined as Re = *ρUL/μ* and describes the ratio of flow inertia to viscous forces; here *U* is the average flow speed, *L* is a characteristic length scale (i.e., height of chamber), and *ρ* and *μ* are the fluid density and viscosity, respectively. Due to the small length scale and low flow rates, the microfluidic device is dominated by linear viscous forces (*Re* < 0.1), which allows for smooth, orderly flow within the chamber. Figure 4B shows that one can produce separate flow streams containing dye and no dye, which means that (at low Re), mRNA molecules in solution will largely move in straight lines parallel to the length of the chamber. Next, we need to control the degree of mixing (or diffusion) among the flow lines. To this end, we need to control the ratio of the rate of diffusion to the rate of advection between each fluid stream. This ratio is characterized by the Peclet number, defined as *Pe* = *UL*/*D*, where *D* is the diffusivity of the RNA molecules (*D* ∼ 5 × 10^−10^ m^2^ s^-1^) and L is a characteristic length scale (i.e., width of chamber^2^/length of chamber). Figure 4B clearly shows that the degree of diffusion between fluid streams decreases as the flow rates is increased, which increases the Pe and Re. Hence, one can expose cells in the microfluidic chamber to a precise mRNA concentration map that is spatially controlled by flow conditions (Re and Pe). This microfluidics device was chosen for use in a subset of studies to illustrate how the TIPeR approach can be made into a high-throughput RNA functional genomics methodology. The device is light addressable (allowing phototransfection in the device) and is designed to permit multiple RNAs to flow across the cells at user defined concentration. With additional development, this approach has the added potential for generating a gradient of RNA concentrations across the device-situated cells, so each cell is phototransfected with different but known amounts of RNA, thereby permitting easy characterization of RNA concentrations/ratios that elicit assayed cellular responses.

The utility of this device for multiple mRNA transfection and biological phenotyping of cells in response to expression of viral proteins is demonstrated by phototransfecting four mRNAs into cultured host 3T3 cells, including those that encode the SARS-CoV-2 Spike protein, Nuc protein, and eGFP (to visualize phototransfected cells). 3T3 cells were used as these cells are often used in high-throughput screening assays (Burbaum and Sigal, 1997; Peña et al., 2015; Flaberg et al., 2011; Sittampalam et al., 1997), hence as this technology is moved to high-throughput use of these cells will provide a wealth of background information that may make such a transition more facile. The inlet protocol was as follows: a saline solution was injected into inlets 1 and 7, an eGFP-encoding mRNA solution was injected into inlets 2 and 6, and a solution containing a mixture of Nuc-encoding mRNA, SARS-CoV-2 Spike protein-encoding mRNA, and CD80-encoding mRNA was injected into inlets 3, 4 and 5. Inlet 3 received the mixture at a concentration of 3.81 μg/mL, whereas inlets 4 and 5 received the mixture at a concentration of 7.63 μg/mL. The position of the parallel fluorescent streams, imaged before each cell phototransfection experiments by confocal imaging of the chamber, allowed for the precise localization of the cells that should express the SARS-CoV-2 proteins. In all experiments the flow rate was set at 3 μL/min for each inlet. After phototransfection, the cover slip was removed from the microfluidic device and incubated for 3 days before measuring the expression levels. The cells were fixed and immunostained with antibodies to the Nucleocapsid, and Spike proteins. The species-specific secondary antibodies that recognize each primary antibody were independently labeled with fluorophores whose excitation spectra can be distinguished from the other fluorophores. eGFP mRNA is introduced at low levels (∼10 molecules/cell) (Sul et al., 2009) to limit the amount of competition for endogenous cellular translation. The Spike protein-encoding mRNA gives a higher level of protein expression than Nuc-encoding mRNA. As observed previously for human astrocytes, the peak mRNA concentration for phototransfection and expression in 3T3 cells is ∼3.4 μg/mL with the drop off at higher concentration not as dramatic as for human astrocyte cells (Figure 4C).

## Discussion

The multi-RNA phototransfection approach described herein has the potential for facilitating a rapid and sustained biological response in light addressable cells. It may be useful in preclinical studies to quickly screen for protein expression dynamics of gene regions from rapidly evolving microorganisms. This may help in preclinical studies designed to identify proteins or protein regions that would be candidates for employing in mRNA-based vaccines. For example, as has been observed for SARS-CoV-2 infection, the viral genome evolves rapidly with many mutations throughout the genome, including the Spike protein-encoding gene to which the original Moderna and Pfizer-BioNTech COVID-19 vaccines were directed. As a consequence, bivalent mRNA COVID-19 vaccines were introduced in 2023 (Lin et al., 2023) that encoded two versions of the Spike protein, one being the original strain and the second being the mutated Spike of Omicron subvariants BA.4 and BA.5. This is in recognition that if a vaccine is made to a single antigen-encoding mRNA, then viruses that have mutated antigens may not be neutralized as well even though an immune response to the initial antigen was developed. While effective, the bivalent vaccine used LNPs as the mRNA delivery molecule so there was little ability to vary the amounts of transfected RNA (Lin et al., 2023). This required extensive pre-clinical work in optimizing LNP chemistry, binding of the mRNAs to the LNPs and selecting a mRNA concentration. Alternatively, as shown herein, using a gradient of RNA abundances, high-throughput single cell phototransfection offers the potential to rapidly titrate the protein expression dynamics of candidate vaccine RNAs. When used in conjunction with animal studies to test the immune response of said concentrations of mRNAs it may be possible to rapidly determine mRNA abundances that optimize the immune response.

In prior work, we showed that transfection of populations of mRNA can transdifferentiate cells (Sul et al., 2009; Kim et al., 2011) and have posited that the ratios of mRNAs that are transfected may be as important or more important than the simple abundances of the mRNAs (Kim J. and Eberwine, 2010). This has started to be explored in assessing the capacity of mRNAs encoding two HIV antigens to elicit an immune response (Zhang et al., 2021). While antibodies to the SARS-CoV-2 Spike protein have proven to be an effective antigen in creating a therapeutic immune response, there are other genic regions that may encode proteins that in conjunction prove to be more potent and provide a wider breadth of immune responses. For example, the Nuc protein which is a structural protein in SARS-CoV-2 virus assembly has higher sequence conservation between virus strains (Dutta et al., 2020). Generating an immune response to the Nuc protein may prove effective against multiple SARS-CoV-2 strains, and when used in concert with Spike may elicit a synergistic response. This has been shown in preclinical studies using LNP delivery of both Spike and Nuc mRNAs (Class et al., 2021; Dangi et al., 2021). This is not surprising as among the most successful vaccines are those using either inactivated or attenuated viruses where much of virus structure remains intact providing more immunogens in a seemingly more natural context. While not compared directly to Spike mRNA vaccines, SARS-CoV-2 attenuated virus vaccines do elicit a humoral and cellular immune response (Hotez and Bottazzi, 2022; Okamura and Ebina, 2021; Khoshnood et al., 2022). However, attenuated viruses also suffer from various side-effects including potential reactivation. This offers the possibility that a cocktail of the mRNAs encoding each of the 6 proteins encoded by the SARS-CoV-2 virus may be a more effective immunogen. This would overcome issues of potential virus reactivation. If this is true, it is possible that there are differing amounts of each protein in the intact virus and the most biologically informative analysis of SARS-CoV-2 intracellular function as well as extracellular immune response, likely requires these ratios of protein abundance to be recapitulated in the infected cells to elicit an optimal response.

The co-mRNA transfection data in Figure 3C show that *in situ* competition in the translation of Spike and Nuc mRNAs, are consistent with previous studies showing that endogenous cellular RNAs compete with each other for resources used in translation including association with RNA binding proteins that are integral to the process of translation (Asselbergs et al., 1978; Godefroy-Colburn and Thach, 1981; Jain et al., 2022; Ray et al., 1983; Schneider-Lunitz et al., 2021). Studies have further shown that SARS-CoV-2 infection results in reduced translation of host-cell mRNAs that have a preponderance of the same codons that are enriched in SARS-CoV-2 (Alonso and Diambra, 2020) also known as epistasis (Mogro et al., 2022; Fumagalli et al., 2023). However, epitasis which would result in the same impact for both RNAs, does not explain the unidirectionality of the observed effect, with the Nuc RNA significantly impacting the translation of Spike mRNA while Spike mRNA has a more limited impact upon Nuc RNA translation. The dominance of Nuc RNA in modulating Spike RNA translation suggests that upon translation of Nuc RNA there is an RNA binding protein (RBP) that is activated that binds to Spike RNA to block its translation. Further the impact of Spike mRNA presence and translation in increasing Nuc immunoreactivity suggests that the translational status of Nuc mRNA is enhanced perhaps by suppressing activity of a Nuc mRNA translation inhibitory RBP through the Spike mRNA competing for its binding. Computational analysis of the primary sequence of SARS-CoV-2 Spike and Nuc RNA sequences shows the presence of RNA sequences that are predicted canonical binding sites for various RBPs (Horlacher et al., 2023). The combination of the translation data presented herein and computational predictions from others (Horlacher et al., 2023) suggests that cells will differ in their ability to harbor or propagate SARS-CoV-2 virus based upon the host cells RBP complement. Further, these data suggest that the amounts and ratios of SARS-CoV-2 protein-encoding mRNAs used in the development of multivalent vaccines may play a role in eliciting optimal cellular responses. The transition from using attenuated microbes with predetermined antigenic profiles to understanding the intricacies of translation within host cells is a hallmark of mRNA vaccines. This shift necessitates a deep dive into the realms of infected cell identities, the diverse landscape of infectious mRNAs, and the nuanced ratios in which they manifest. With mRNA vaccines, the focus shifts from using attenuated microbes with their pre-defined antigenic profiles to understanding the competition for translation capacity within the host cells. This shift involves considering factors such as the identity of infected cells, the diversity of infectious mRNAs, and the intricate ratios in which they are expressed.

As a general discovery tool, the simultaneous use of multiple protein encoding mRNAs may speed up vaccine development. Often it is difficult to know what protein or antigen site on a protein of an invading microbe will prove to be most useful in generating protective immune responses. Identification of the best antigenic regions can be hastened by taking a “shot gun” multigenic or multiantigen transfection approach where multiple mRNAs encoding genic regions from the foreign entity are co-transfected into cells and their expression dynamics used to identify ratios of mRNAs that provide the most cell surface expression. Further, querying the single cell transcriptome of SARS-CoV-2 infected cells to determine the relative abundances of the mRNAs expressed from the viral genome so that these same abundances can be phototransfected into cells in the same ratio as expressed upon infection to mimic a cells normal expression of the virus and consequent antigen presentation as would be the case in immunization with inactivated or attenuated virus (Xia et al., 2020). This would enable the “normal” systems biology of SARS-CoV-2 infection to drive the cellular expression of antigens and may be instructive of the organismal immune response.

While phototransfection of mRNA is unlikely to be tenable as a means for widespread immunization, its use can be envisioned to alter a cell’s biology locally and transiently in specialized applications where light-assisted examination, manipulation or remediation is performed. Co-expression of multiple phototransfected mRNAs in single user identified cells, including dispersed human astrocytes in primary cell culture, illustrates the potential for expressing proteins in cells of particular phenotypes. For example, given that SARS-CoV-2 infects human astrocytes of the central nervous system (Crunfli et al., 2021; Andrews et al., 2022), it may be preferable to express multiple SARS-CoV-2 gene products in single astrocytes rather than other cell types, to assess the biological impact of their expression upon host cell biology. The recipient cell type is an important factor in dictating the amount of translation from transfected RNA (Malone et al., 1989; Kim T. K. and Eberwine, 2010; Kowalski et al., 2019). While human iPSC cell lines that give rise to astrocytes have been developed, the use of unmodified human cells has potential for eliminating any confounds introduced due to the immortalization and differentiation of the cells into astrocytes. Since TIPeR (transcriptome induced phenotype remodeling) is a highly scalable single cell technology, getting enough cells to perform properly powered experimental manipulations of difficult to acquire cells such as human neuronal cells become less problematic. Phototransfection promises to enable the high-throughput analysis of multiple mRNAs at varying abundances to assess the systems biological analysis of virus infection.

## Conclusion

The ability to assess the cell biologies induced by expression of multiple mRNAs has been technologically challenging. Data presented in this paper show that SARS-CoV 2 encoded mRNAs can compete for translation in human astrocytes. The ability to assess RNA mediated biologies in primary cell cultures of human astrocytes shows that such cells are amenable to omics manipulation and may be useful in personalized therapeutic development. Further, these results show that in using multiple mRNAs to generate antigens to elicit an immune response, that consideration of the amounts of the transfected RNAs is required to insure optimal amounts of translated proteins. This was discernible because the TIPeR approach permits titration of the amount of RNA that is introduced into the cell so that RNA concentration dependent outcomes can be quantified. The high-throughput TIPeR approach is amenable to analysis of the expression of any number of RNAs in user defined abundances in selected cells. This is possible as phototransfection can be repeated multiple times on the same cell. This approach provides a functional genomics approach that is free of over-expression artifacts often introduced by promoter driven transgenes. It is anticipated that functional analysis of multiple RNAs that are introduced into cells in user defined abundance will be useful in manipulating disease-associated clinically relevant KEGG- or SNAP-defined pathways (Kanehisa et al., 2017; Agrawal et al., 2018) or Go designated functionality (Balakrishnan et al., 2013).

## Methods and materials

### mRNA *in vitro* transcription

Optimized mRNAs encoding SARS-CoV-2 Spike and Nucleocapsid proteins are made from plasmid DNA templates as described previously (Laczkó et al., 2020) Briefly, SARS-CoV-2 gene fragments were synthesized (GenScript) and cloned into an mRNA production plasmid. mRNAs are produced using T7 RNA polymerase on linearized plasmids. mRNAs were transcribed to contain 101 nucleotide-long poly(A) tails. Capping of the *in vitro* transcribed mRNAs will be performed co-transcriptionally using the trinucleotide cap1 analog, CleanCap (TriLink). mRNAs were purified by cellulose purification, as described (Baiersdörfer et al., 2019). All mRNAs were analyzed by agarose gel electrophoresis and stored frozen at −20°C until use.

### Human primary cell cultures

The resected human cortical brain tissue was immediately placed in ice-cold aCSF and transported to the laboratory for slicing using a VT1200S vibratome (Leica). Excessive blood clots were carefully removed, and white matter was trimmed before slicing 100–150 μm thick sections in ice-cold sucrose cutting solution (248 mM sucrose, 1 mM KCl, 26 mM NaHCO_3_, 10 mM glucose, 1 mM CaCl_2_, and 10 mM MgCl_2_; bubbled with 95% O_2_ and 5% CO_2_). Each section was then transferred to a resting chamber with normal aCSF (124 mM NaCl, 4 mM KCl, 26 mM NaHCO_3_, 10 mM glucose, 2 mM CaCl_2_, and 2 mM MgCl_2_; bubbled with 95% O_2_ and 5% CO_2_) and allowed to rest for at least 30 min. Primary cultures were established by enzymatic dissociation using papain (Worthington Biochemicals) followed by gentle mechanical dissociation with a fire-polished glass Pasteur pipette. The cells were counted using an Autocounter (Invitrogen) and plated at a density of 1.2 × 10^5^ cells/mL on poly-D-lysine and laminin-coated gridded Petri dishes (80 μg/mL PDL; Sigma P6407, 1 g/mL laminin; Corning #354239, Grid-500). The cultures were maintained in DMEM (#10-013, Corning) supplemented with 10% FBS (#100-500, Gemcell), 2.5% B-27 (#17504-044, Gibco), and 1% penicillin-streptomycin (#17-602E, Lonza) at CO_2_ incubator (37°C, 95% humidity, and 5% CO_2_). The culture medium was replaced every 2–3 days with fresh media (50% replacement).

### PC12 and 3T3 cell cultures

The cell lines PC12 Adh (CRL-1721.1) and 3T3 (CRL-1658) were obtained from ATCC and cultured according to the provided protocol. Each cell line was expanded up to passage 3 and aliquoted for later use. To minimize variability in gene expression due to passaging, experiments were performed between passage 5 and 7. The defrosted cells were plated and cultured in T25 flasks at 37°C, 95% humidity, and 5% CO_2_ in the following media: for PC12 cells, F-12K (#30-2004; ATCC), FBS (#30-2020; ATCC) 2.5%, and penicillin-streptomycin (#17-602E; Lonza) 1%; for 3T3 cells, DMEM (#10-013; Corning), BCS (#30-2020; ATCC) 1%, and penicillin-streptomycin (#17-602E, Lonza) 1%. The medium was changed every 2–3 days by replacing 50% with fresh medium. For experiments, cells were enzymatically detached from T25 flasks (using 2.5% trypsin, #15090-046; Gibco), mechanically dissociated with a fire-polished glass Pasteur pipette and plated onto gridded chambers at a density of 1.5 × 10^5^ cells/mL live cells (using the same plate coating conditions as in human cases). Live cell density for plating was determined using automated counting with trypan blue (1%, Sigma-Aldrich). No mycoplasm contamination was in the continuous cell line or primary cell cultures.

### Phototransfection

Phototransfection was used to introduce mRNAs into targeted cells. Individual cells were located on gridded Petri dishes to assess the method’s effects. The high-power femtosecond IR laser (Mai-Tai, Spectral-physics) at a wavelength of 700 nm was irradiated onto the cells using a Zeiss 710 Meta upright laser-scanning microscope equipped with a 40x water immersion lens (W-Plan Apochromatic, N.A., 1.0). The IR laser power was set to 30 mW at the back aperture of the lens, and the power was adjusted to 15% based on the cell type and conditions. The power adjustment was performed for each batch of cells to assess phototransfection efficiency by measuring the transient fluorescent signal increase using a membrane-impermeable fluorescent dye, fluorescein (Sigma-Aldrich), from the bath solution to the cytoplasm of the cells. The phototransfection site on the cells was determined using transmitted light gradient contrast imaging from a 488 nm argon laser. The IR laser beam was focused as 3 × 3 pixels (0.42μm/pixel, 177.32 msec dwell time/pixel) and irradiated six times with a time interval of 1 s. The cells in the Petri dish were perfused with RNAse-free extracellular saline (140 mM NaCl, 5.4mM KCl, 1mM MgCl_2_, 2 mM CaCl_2_, 16 mM glucose, 10 mM HEPES, adjusted to pH 7.3), and the target mRNAs were introduced just before phototransfection. Immediately after phototransfection, the Petri dish was moved to a biosafety hood, rinsed three times with sterile HBSS, and transferred to an incubator for subsequent processing. The viability of the phototransfected cells was confirmed with phase contrast microscopy 6 hours later, and under the given settings, the viability was over 61.5% (PC12).

### Immunocytochemical analysis of antigen presentation

Cells were washed with Dulbecco’s phosphate buffered saline (DPBS) and fixed with 4% paraformaldehyde (PFA) in DPBS buffer at room temperature for 15 min followed by three washes in DPBS. The residual PFA was quenched by incubation the cells in 0.2M glycine in DPBS for 5 min. Cells were then washed with DBPS for three times and permeabolized with 0.1% Triton X-100 in DPBS for 5 min. After three DPBS washes, cells were blocked with 5% BSA in PBST (0.1% Tween20 in DPBS) for 1 h at room temperature. Cells were then washed with PBST for 3 times, each time for 5 min. Primary antibodies were diluted in 3% BSA in PBST and incubated with cells at 4° overnight. After five washes with PBST, each time for 5 min, cells were then incubated with the diluted secondary antibodies in 3% BSA in PBST at 37° for 1 h. The cells were washed with PBST for five times, each time for 5 min, followed by one wash in distilled water. After totally dried in the room temperature, cells were mounted with Fluoromount-G (catalog no. 0100-01, Southern Biotech). The outcomes were evaluated by confocal microscopy.

Primary antibodies used for the immunofluorescence analysis include mouse anit-SARS-CoV-2 (COVID-19) Spike antibody [1A9] (catalog no. GTX632604, GeneTex; 1:1,000 dilution), rabbit anti-SARS-CoV-2 Nuc protein antibody (catalog no. ab273167, Abcam; 1:2500 dilution), rabbit anti-CD86 antibody [EPR21962] (catalog no. ab239075, Abcam; 1:3000 dilution), and goat anti-CD80 polyclonal antibody (catalog no. PA5-19211, ThermoFisher; 1:1,000 dilution). Secondary antibodies used include Alexa Fluor 405 Donkey anti-Goat IgG (catalog no. A48259, Invitrogen; 1:500 dilution), Alexa Fluor 546 Goat anti-mouse IgG (catalog no. A11030, Invitrogen; 1:1,000 dilution), and Alexa Fluor 633 Goat anti-Rabbit IgG (catalog no. A21071, Invitrogen; 1:1,000 dilution). Samples were imaged using a Zeiss 880 Meta upright laser-scanning confocal microscope equipped with a 40x lens (W-Plan Apochromatic, N.A., 1.0) with a resolution of 1024 × 1024 pixels (212 × 212 μm^2^) and a pixel dwell time of 1.58 μsec/pixel at a 1 airy unit pinhole size. The spectra of each Alexa dye were separated by pairs of 405 and 488/561/633 dichroic mirrors, and corresponding fluorescence signals were sequentially measured for Alexa405 (ex405nm/em410-489 nm), Alexa546 (ex561/em546-638 nm), and Alexa633 (ex633/em638-747 nm) to minimize fluorescence signal bleeding between channels. All images were processed using the MetaMorph Offline version 7.8 image processing software from quantify the fluorescence signals.

### Device design and fabrication

The reversibly sealable microfluidic device was fabricated using standard soft-lithography techniques. In brief, the mold of the bottom layer of the device was fabricated by spin-coating a negative photoresist (SU-8 2050, Microchem) on a silicon wafer to obtain 150 um tall microfluidic channels. The rectangular chamber on the bottom layer was 20 mm long by 9.5 mm wide. PDMS (Sylgard 184, Dow Corning) was cast on to the prepared bottom layer mold and extracted from the mold. The mold of the top layer of the device was created by bonding a 12 mm by 22 mm glass coverslip to a glass slide using an optical adhesive and forming a raised border around the glass slide using aluminum foil. PDMS (Sylgard 184, Dow Corning) was cast on to the prepared top layer mold and two slabs of PDMS were cut out from the mold. Seven inlets and an outlet were punched in the cured PDMS cast of the top layer pieces. The bottom layer received a thin layer of PDMS cross-linking agent and the two top layer PDMS slabs were brought into contact with the bottom layer. The resulting device was cured in a 75C oven for at least 30 min to ensure a secure bond between the layers. The microfluidic device was designed to allow for a 200 μm glass cover slip to be inserted into the pockets that exist between the top and bottom PDMS layers without leaking at flow rates up to 350 μL/min.

### Device flow methods

Prior to each experiment, the PDMS chamber was immersed in a saline solution (DPBS) and degassed for 10 min to avoid bubble entrapment in the microchannels that might disturb the flow pattern. The cover slip cultured with cells was inserted above the chamber of the bottom PDMS layer with saline added above the chamber prior to insertion to prevent damage to the cells. The device was then mounted on a flat platform containing two magnets. A magnetic metallic frame was placed above the cover slip to tightly secure the edges of the cover slip to the bottom PDMS layer. The fluids were contained in 500 uL syringes and the flow rate as controlled by a syringe pump (Harvard Apparatus PhD). Each of the seven syringes were connected by 26-gauge PTFE tubing (Hamilton) to the device inlets. The outlet was connected to 0.034″ ID X 0.052” OD polyethylene tubing set a lower height than the device. This positioning was designed to avoid hydrostatic pressure built up at the outlet that could compromise the leak-proof nature of the device.

### Statistical analysis

Data are presented as mean ± SEM. For all experiments, data normality was first assessed using a Shapiro-Wilk normality test. For normally distributed data, differences between groups were evaluated by unpaired two-tailed t-tests or one-way ANOVA with Dunnett’s multiple comparisons *post hoc* tests. Sigmaplot 12.0 was used for these analyses and prism 9 was used for creating the plots.

## Data Availability

The original contributions presented in the study are included in the article/Supplementary Material, further inquiries can be directed to the corresponding author.

## Supplementary material

SUPPLEMENTARY FIGURE S1 Optimization of phototransfection parameters. Since phototransfection depends upon laser introduction of transient pores into a cell membrane so that RNA can diffuse in, various parameters need to be optimized each time phototransfection is performed. **(A)** Among the high-power multi-photon laser parameters that were optimized were, wavelength, power, and irradiation duration all done by using the membrane-impermeable fluorescent dye fluorescein on live 3T3 cells as an indicator of successful phototransfection. Fluorescein (100 μM) was applied in the extracellular solution and upon successful laser irradiation focused at 6 μm above the cell membrane transient pores formed and fluorescein diffused into the cell such that fluorescence is visible inside the cell. The cell membrane is outlined with the white dashed line. **(B)** However, excessive power resulting from misfocused irradiation directly on the plasma membrane can cause excessive dye influx due to failure in resealing the pores, leading to cell death, as indicated by fluorescein accumulation in mitochondria in the indicated cell that is in the process of dying. **(C)** Accumulation of fluorescence in the nucleus due to improper laser focus is also an indicator of cell death. Scale bar is 20 microns. Color Scale shows the intensity of fluorescence signal in arbitrary units with the maximum value indicated in red.

## References

[B1] Agrawal M. Zitnik M. Leskovec J. (2018). Large-scale analysis of disease pathways in the human interactome. Pac. Symp. Biocomput. Pac. Symp. Biocomput. 23, 111–122. 10.1142/9789813235533_0011 29218874 PMC5731453

[B2] Alameh M.-G. Tombácz I. Bettini E. Lederer K. Sittplangkoon C. Wilmore J. R. (2021). Lipid nanoparticles enhance the efficacy of mRNA and protein subunit vaccines by inducing robust T follicular helper cell and humoral responses. Immunity 54, 2877–2892.e7. 10.1016/j.immuni.2021.11.001 34852217 PMC8566475

[B3] Alonso A. M. Diambra L. (2020). SARS-CoV-2 codon usage bias downregulates host expressed genes with similar codon usage. Front. Cell Dev. Biol. 8, 831. 10.3389/fcell.2020.00831 32974353 PMC7468442

[B4] Andrews M. G. Mukhtar T. Eze U. C. Simoneau C. R. Ross J. Parikshak N. (2022). Tropism of SARS-CoV-2 for human cortical astrocytes. Proc. Natl. Acad. Sci. 119, e2122236119. 10.1073/pnas.2122236119 35858406 PMC9335272

[B5] Angel M. Yanik M. F. (2010). Innate immune suppression enables frequent transfection with RNA encoding reprogramming proteins. PLOS ONE 5, e11756. 10.1371/journal.pone.0011756 20668695 PMC2909252

[B6] Arevalo C. P. Bolton M. J. Le Sage V. Ye N. Furey C. Muramatsu H. (2022). A multivalent nucleoside-modified mRNA vaccine against all known influenza virus subtypes. Science 378, 899–904. 10.1126/science.abm0271 36423275 PMC10790309

[B7] Asselbergs F. A. M. Peters W. H. M. van Venrooij W. J. Bloemendal H. (1978). The effect of the messenger RNA concentration on the competitive inhibition of translation by cap-analogues. Mol. Biol. Rep. 4, 177–180. 10.1007/BF00777520 739985

[B8] Baden L. R. Sahly H. M. E. Essink B. Kotloff K. Frey S. Novak R. (2020). Efficacy and safety of the mRNA-1273 SARS-CoV-2 vaccine. N. Engl. J. Med. 10.1056/NEJMoa2035389 PMC7787219 33378609

[B9] Baiersdörfer M. Boros G. Muramatsu H. Mahiny A. Vlatkovic I. Sahin U. (2019). A facile method for the removal of dsRNA contaminant from *in vitro*-Transcribed mRNA. Mol. Ther. - Nucleic Acids 15, 26–35. 10.1016/j.omtn.2019.02.018 30933724 PMC6444222

[B10] Balakrishnan R. Harris M. A. Huntley R. Van Auken K. Cherry J. M. (2013). A guide to best practices for Gene Ontology (GO) manual annotation. Database J. Biol. Databases Curation 2013, bat054. 10.1093/database/bat054 PMC3706743 23842463

[B11] Barrett L. E. Sul J.-Y. Takano H. Van Bockstaele E. J. Haydon P. G. Eberwine J. H. (2006). Region-directed phototransfection reveals the functional significance of a dendritically synthesized transcription factor. Nat. Methods 3, 455–460. 10.1038/nmeth885 16721379

[B12] Billingsley M. M. Singh N. Ravikumar P. Zhang R. June C. H. Mitchell M. J. (2020). Ionizable lipid nanoparticle-mediated mRNA delivery for human CAR T cell engineering. Nano Lett. 20, 1578–1589. 10.1021/acs.nanolett.9b04246 31951421 PMC7313236

[B13] Brouwer P. J. M. Brinkkemper M. Maisonnasse P. Dereuddre-Bosquet N. Grobben M. Claireaux M. (2021). Two-component spike nanoparticle vaccine protects macaques from SARS-CoV-2 infection. Cell 184, 1188–1200. 10.1016/j.cell.2021.01.035 33577765 PMC7834972

[B14] Burbaum J. J. Sigal N. H. (1997). New technologies for high-throughput screening. Curr. Opin. Chem. Biol. 1, 72–78. 10.1016/s1367-5931(97)80111-1 9667842

[B15] Burrell C. J. Howard C. R. Murphy F. A. (2017). “Vaccines and vaccination,” in Fenner and white’s medical virology (Elsevier), 155–167. Available at: https://linkinghub.elsevier.com/retrieve/pii/B9780123751560000114.

[B16] Chen M. Ma Y. Chang W. (2022). SARS-CoV-2 and the nucleus. Int. J. Biol. Sci. 18, 4731–4743. 10.7150/ijbs.72482 35874947 PMC9305274

[B17] Class J. Dangi T. Richner J. M. Penaloza-MacMaster P. (2021). A SARS CoV-2 nucleocapsid vaccine protects against distal viral dissemination. preprint, Immunology. 10.1101/2021.04.26.440920 PMC8367759 34450033

[B18] Crunfli F. Carregari V. C. Veras F. P. Vendramini P. H. Valença A. G. F. Antunes A. S. L. M. (2021). SARS-CoV-2 infects brain astrocytes of COVID-19 patients and impairs neuronal viability. medRxiv. 10.1101/2020.10.09.20207464

[B19] Dangi T. Class J. Palacio N. Richner J. M. MacMaster P. P. (2021). Combining spike- and nucleocapsid-based vaccines improves distal control of SARS-CoV-2. Cell Rep. 36, 109664. 10.1016/j.celrep.2021.109664 34450033 PMC8367759

[B20] Diener Y. Jurk M. Kandil B. Choi Y.-H. Wild S. Bissels U. (2015). RNA-based, transient modulation of gene expression in human haematopoietic stem and progenitor cells. Sci. Rep. 5, 17184. 10.1038/srep17184 26599627 PMC4657003

[B21] Dutta N. K. Mazumdar K. Gordy J. T. (2020). The nucleocapsid protein of SARS–CoV-2: a target for vaccine development. J. Virol. 94, 006477–e720. 10.1128/JVI.00647-20 PMC7307180 32546606

[B22] Flaberg E. Markasz L. Petranyi G. Stuber G. Dicső F. Alchihabi N. (2011). High-throughput live-cell imaging reveals differential inhibition of tumor cell proliferation by human fibroblasts. Int. J. Cancer 128, 2793–2802. 10.1002/ijc.25612 20715102

[B23] Freyn A. W. Ramos da Silva J. Rosado V. C. Bliss C. M. Pine M. Mui B. L. (2020). A multi-targeting, nucleoside-modified mRNA influenza virus vaccine provides broad protection in mice. Mol. Ther. J. Am. Soc. Gene Ther. 28, 1569–1584. 10.1016/j.ymthe.2020.04.018 PMC7335735 32359470

[B24] Fumagalli S. E. Padhiar N. H. Meyer D. Katneni U. Bar H. DiCuccio M. (2023). Analysis of 3.5 million SARS-CoV-2 sequences reveals unique mutational trends with consistent nucleotide and codon frequencies. Virol. J. 20, 31. 10.1186/s12985-023-01982-8 36812119 PMC9936480

[B25] Godefroy-Colburn T. Thach R. E. (1981). The role of mRNA competition in regulating translation. IV. Kinetic model. J. Biol. Chem. 256, 11762–11773. 10.1016/s0021-9258(19)68471-1 7298630

[B26] Grifoni A. Weiskopf D. Ramirez S. I. Mateus J. Dan J. M. Moderbacher C. R. (2020). Targets of T Cell responses to SARS-CoV-2 coronavirus in humans with COVID-19 disease and unexposed individuals. Cell 181, 1489–1501. 10.1016/j.cell.2020.05.015 32473127 PMC7237901

[B27] Guerrini G. Magrì D. Gioria S. Medaglini D. Calzolai L. (2022). Characterization of nanoparticles-based vaccines for COVID-19. Nat. Nanotechnol. 17, 570–576. 10.1038/s41565-022-01129-w 35710950

[B28] Hogan M. J. Pardi N. (2022). mRNA vaccines in the COVID-19 pandemic and beyond. Annu. Rev. Med. 73, 17–39. 10.1146/annurev-med-042420-112725 34669432

[B29] Horlacher M. Oleshko S. Hu Y. Ghanbari M. Cantini G. Schinke P. (2023). A computational map of the human-SARS-CoV-2 protein–RNA interactome predicted at single-nucleotide resolution. Nar. Genomics Bioinforma. 5, lqad010. 10.1093/nargab/lqad010 PMC9940458 36814457

[B30] Hotez P. J. Bottazzi M. E. (2022). Whole inactivated virus and protein-based COVID-19 vaccines. Annu. Rev. Med. 73, 55–64. 10.1146/annurev-med-042420-113212 34637324

[B31] Hu B. Guo H. Zhou P. Shi Z.-L. (2021). Characteristics of SARS-CoV-2 and COVID-19. Nat. Rev. Microbiol. 19, 141–154. 10.1038/s41579-020-00459-7 33024307 PMC7537588

[B32] Huang S. Fishell G. (2022). SARS-CoV-2, astrocytes are in it for the long haul. Proc. Natl. Acad. Sci. 119, e2209130119. 10.1073/pnas.2209130119 35858460 PMC9335203

[B33] Jain A. Margaliot M. Gupta A. K. (2022). Large-scale mRNA translation and the intricate effects of competition for the finite pool of ribosomes. J. R. Soc. Interface 19, 20220033. 10.1098/rsif.2022.0033 35259953 PMC8922411

[B34] Kallen K.-J. Theß A. (2014). A development that may evolve into a revolution in medicine: mRNA as the basis for novel, nucleotide-based vaccines and drugs. Ther. Adv. Vaccines 2, 10–31. 10.1177/2051013613508729 24757523 PMC3991152

[B35] Kanehisa M. Furumichi M. Tanabe M. Sato Y. Morishima K. (2017). KEGG: new perspectives on genomes, pathways, diseases and drugs. Nucleic Acids Res. 45, D353–D361. 10.1093/nar/gkw1092 27899662 PMC5210567

[B36] Khorkova O. Stahl J. Joji A. Volmar C.-H. Wahlestedt C. (2023). Amplifying gene expression with RNA-targeted therapeutics. Nat. Rev. Drug Discov. 22, 539–561. 10.1038/s41573-023-00704-7 37253858 PMC10227815

[B37] Khoshnood S. Arshadi M. Akrami S. Koupaei M. Ghahramanpour H. Shariati A. (2022). An overview on inactivated and live-attenuated SARS-CoV-2 vaccines. J. Clin. Lab. Anal. 36, e24418. 10.1002/jcla.24418 35421266 PMC9102488

[B38] Kim J. Eberwine J. (2010). RNA: state memory and mediator of cellular phenotype. Trends Cell Biol. 20, 311–318. 10.1016/j.tcb.2010.03.003 20382532 PMC2892202

[B39] Kim T. K. Eberwine J. H. (2010). Mammalian cell transfection: the present and the future. Anal. Bioanal. Chem. 397, 3173–3178. 10.1007/s00216-010-3821-6 20549496 PMC2911531

[B40] Kim T. K. Sul J.-Y. Peternko N. B. Lee J. H. Lee M. Patel V. V. (2011). Transcriptome transfer provides a model for understanding the phenotype of cardiomyocytes. Proc. Natl. Acad. Sci. U. S. A. 108, 11918–11923. 10.1073/pnas.1101223108 21730152 PMC3141937

[B41] Kowalski P. S. Rudra A. Miao L. Anderson D. G. (2019). Delivering the messenger: Advances in technologies for therapeutic mRNA delivery. Mol. Ther. 27, 710–728. 10.1016/j.ymthe.2019.02.012 30846391 PMC6453548

[B42] Kulkarni J. A. Witzigmann D. Thomson S. B. Chen S. Leavitt B. R. Cullis P. R. (2021). The current landscape of nucleic acid therapeutics. Nat. Nanotechnol. 16, 630–643. 10.1038/s41565-021-00898-0 34059811

[B43] Laczkó D. Hogan M. J. Toulmin S. A. Hicks P. Lederer K. Gaudette B. T. (2020). A single immunization with nucleoside-modified mRNA vaccines elicits strong cellular and humoral immune responses against SARS-CoV-2 in mice. Immunity 53, 724–732. 10.1016/j.immuni.2020.07.019 32783919 PMC7392193

[B44] Leitner W. W. Ying H. Restifo N. P. (1999). DNA and RNA-based vaccines: principles, progress and prospects. Vaccine 18, 765–777. 10.1016/s0264-410x(99)00271-6 10580187 PMC1986720

[B45] Li Y.-D. Chi W.-Y. Su J.-H. Ferrall L. Hung C.-F. Wu T.-C. (2020). Coronavirus vaccine development: from SARS and MERS to COVID-19. J. Biomed. Sci. 27, 104. 10.1186/s12929-020-00695-2 33341119 PMC7749790

[B46] Lin D.-Y. Xu Y. Gu Y. Zeng D. Wheeler B. Young H. (2023). Effectiveness of bivalent boosters against severe Omicron infection. N. Engl. J. Med. 388, 764–766. 10.1056/NEJMc2215471 36734847 PMC9933929

[B47] Malone R. W. Felgner P. L. Verma I. M. (1989). Cationic liposome-mediated RNA transfection. Proc. Natl. Acad. Sci. 86, 6077–6081. 10.1073/pnas.86.16.6077 2762315 PMC297778

[B48] McMahon M. O’Dell G. Tan J. Sárközy A. Vadovics M. Carreño J. M. (2022). Assessment of a quadrivalent nucleoside-modified mRNA vaccine that protects against group 2 influenza viruses. Proc. Natl. Acad. Sci. U. S. A. 119, e2206333119. 10.1073/pnas.2206333119 36322769 PMC9659346

[B49] Miao L. Lin J. Huang Y. Li L. Delcassian D. Ge Y. (2020). Synergistic lipid compositions for albumin receptor mediated delivery of mRNA to the liver. Nat. Commun. 11, 2424. 10.1038/s41467-020-16248-y 32415122 PMC7229004

[B50] Moghimi S. M. (2021). Allergic reactions and anaphylaxis to LNP-based COVID-19 vaccines. Mol. Ther. 29, 898–900. 10.1016/j.ymthe.2021.01.030 33571463 PMC7862013

[B51] Mogro E. G. Bottero D. Lozano M. J. (2022). Analysis of SARS-CoV-2 synonymous codon usage evolution throughout the COVID-19 pandemic. Virology 568, 56–71. 10.1016/j.virol.2022.01.011 35134624 PMC8808327

[B52] Moradian H. Roch T. Lendlein A. Gossen M. (2020). mRNA transfection-induced activation of primary human monocytes and macrophages: dependence on carrier system and nucleotide modification. Sci. Rep. 10, 4181. 10.1038/s41598-020-60506-4 32144280 PMC7060354

[B53] Oberli M. A. Reichmuth A. M. Dorkin J. R. Mitchell M. J. Fenton O. S. Jaklenec A. (2017). Lipid nanoparticle assisted mRNA delivery for potent cancer immunotherapy. Nano Lett. 17, 1326–1335. 10.1021/acs.nanolett.6b03329 28273716 PMC5523404

[B54] Okamura S. Ebina H. (2021). Could live attenuated vaccines better control COVID-19? Vaccine 39, 5719–5726. 10.1016/j.vaccine.2021.08.018 34426024 PMC8354792

[B55] Pardi N. Carreño J. M. O’Dell G. Tan J. Bajusz C. Muramatsu H. (2022). Development of a pentavalent broadly protective nucleoside-modified mRNA vaccine against influenza B viruses. Nat. Commun. 13, 4677. 10.1038/s41467-022-32149-8 35945226 PMC9362976

[B56] Pardi N. Hogan M. J. Porter F. W. Weissman D. (2018). mRNA vaccines — a new era in vaccinology. Nat. Rev. Drug Discov. 17, 261–279. 10.1038/nrd.2017.243 29326426 PMC5906799

[B57] Pardi N. Tuyishime S. Muramatsu H. Kariko K. Mui B. L. Tam Y. K. (2015). Expression kinetics of nucleoside-modified mRNA delivered in lipid nanoparticles to mice by various routes. J. Control. Release Off. J. Control. Release Soc. 217, 345–351. 10.1016/j.jconrel.2015.08.007 PMC4624045 26264835

[B58] Pardi N. Weissman D. (2017). Nucleoside modified mRNA vaccines for infectious diseases. Methods Mol. Biol. Clifton N. J. 1499, 109–121. 10.1007/978-1-4939-6481-9_6 27987145

[B59] Pascolo S. (2004). Messenger RNA-based vaccines. Expert Opin. Biol. Ther. 4, 1285–1294. 10.1517/14712598.4.8.1285 15268662

[B60] Peña I. Pilar Manzano M. Cantizani J. Kessler A. Alonso-Padilla J. Bardera A. I. (2015). New compound sets identified from high throughput phenotypic screening against three kinetoplastid parasites: an open resource. Sci. Rep. 5, 8771. 10.1038/srep08771 25740547 PMC4350103

[B61] Qin S. Tang X. Chen Y. Chen K. Fan N. Xiao W. (2022). mRNA-based therapeutics: powerful and versatile tools to combat diseases. Signal Transduct. Target. Ther. 7, 166–235. 10.1038/s41392-022-01007-w 35597779 PMC9123296

[B62] Qureischi M. Mohr J. Arellano-Viera E. Knudsen S. E. Vohidov F. Garitano-Trojaola A. (2022). “Chapter One - mRNA-based therapies: preclinical and clinical applications,” in International Review of Cell and molecular biology. Editors Aranda F. Berraondo P. Galluzzi L. (Academic Press), 1–54. https://www.sciencedirect.com/science/article/pii/S1937644822000521)vol.372ofmRNA-BasedTherapeutics. 10.1016/bs.ircmb.2022.04.007 36064262

[B63] Raes L. Stremersch S. Fraire J. C. Brans T. Goetgeluk G. De Munter S. (2020). Intracellular delivery of mRNA in adherent and suspension cells by vapor nanobubble photoporation. Nano-Micro Lett. 12, 185. 10.1007/s40820-020-00523-0 PMC7770675 34138203

[B64] Ray B. K. Brendler T. G. Adya S. Daniels-McQueen S. Miller J. K. Hershey J. W. (1983). Role of mRNA competition in regulating translation: further characterization of mRNA discriminatory initiation factors. Proc. Natl. Acad. Sci. U. S. A. 80, 663–667. 10.1073/pnas.80.3.663 6572361 PMC393439

[B65] Reichmuth A. M. Oberli M. A. Jaklenec A. Langer R. Blankschtein D. (2016). mRNA vaccine delivery using lipid nanoparticles. Ther. Deliv. 7, 319–334. 10.4155/tde-2016-0006 27075952 PMC5439223

[B66] Robinson E. MacDonald K. D. Slaughter K. McKinney M. Patel S. Sun C. (2018). Lipid nanoparticle-delivered chemically modified mRNA restores chloride secretion in cystic fibrosis. Mol. Ther. J. Am. Soc. Gene Ther. 26, 2034–2046. 10.1016/j.ymthe.2018.05.014 PMC6094356 29910178

[B67] Sajid A. Matias J. Arora G. Kurokawa C. DePonte K. Tang X. (2021). mRNA vaccination induces tick resistance and prevents transmission of the Lyme disease agent. Sci. Transl. Med. 13, eabj9827. 10.1126/scitranslmed.abj9827 34788080

[B68] Sattar S. Kabat J. Jerome K. Feldmann F. Bailey K. Mehedi M. (2023). Nuclear translocation of spike mRNA and protein is a novel feature of SARS-CoV-2. Front. Microbiol. 14, 1073789. 10.3389/fmicb.2023.1073789 36778849 PMC9909199

[B69] Schneider-Lunitz V. Ruiz-Orera J. Hubner N. van Heesch S. (2021). Multifunctional RNA-binding proteins influence mRNA abundance and translational efficiency of distinct sets of target genes. PLOS Comput. Biol. 17, e1009658. 10.1371/journal.pcbi.1009658 34879078 PMC8687540

[B70] Schoenmaker L. Witzigmann D. Kulkarni J. A. Verbeke R. Kersten G. Jiskoot W. (2021). mRNA-lipid nanoparticle COVID-19 vaccines: structure and stability. Int. J. Pharm. 601, 120586. 10.1016/j.ijpharm.2021.120586 33839230 PMC8032477

[B71] Shirbhate E. Pandey J. Patel V. K. Kamal M. Jawaid T. Gorain B. (2021). Understanding the role of ACE-2 receptor in pathogenesis of COVID-19 disease: a potential approach for therapeutic intervention. Pharmacol. Rep. 73, 1539–1550. 10.1007/s43440-021-00303-6 34176080 PMC8236094

[B72] Sittampalam G. S. Kahl S. D. Janzen P. (1997). High-throughput screening: advances in assay technologies, Curr Opin Chem Biol. 1, 384-91. 10.1016/s1367-5931(97)80078-6 9667878

[B73] Song E. Zhang C. Israelow B. Lu-Culligan A. Prado A. V. Skriabine S. (2021). Neuroinvasion of SARS-CoV-2 in human and mouse brain. J. Exp. Med. 218, e20202135. 10.1084/jem.20202135 33433624 PMC7808299

[B74] Spaethling J. M. Na Y.-J. Lee J. Ulyanova A. V. Baltuch G. H. Bell T. J. (2017). Primary cell culture of live neurosurgically resected aged adult human brain cells and single cell transcriptomics. Cell Rep. 18, 791–803. 10.1016/j.celrep.2016.12.066 28099855 PMC5316103

[B75] Sul J.-Y. Wu C. K. Zeng F. Jochems J. Lee M. T. Kim T. K. (2009). Transcriptome transfer produces a predictable cellular phenotype. Proc. Natl. Acad. Sci. 106, 7624–7629. 10.1073/pnas.0902161106 19380745 PMC2670883

[B76] Thanh Le T. Andreadakis Z. Kumar A. Gómez Román R. Tollefsen S. Saville M. (2020). The COVID-19 vaccine development landscape. Nat. Rev. Drug Discov. 19, 305–306. 10.1038/d41573-020-00073-5 32273591

[B77] Turner J. S. O’Halloran J. A. Kalaidina E. Kim W. Schmitz A. J. Zhou J. Q. (2021). SARS-CoV-2 mRNA vaccines induce persistent human germinal centre responses. Nature 596, 109–113. 10.1038/s41586-021-03738-2 34182569 PMC8935394

[B78] Verbeke R. Lentacker I. De Smedt S. Dewitte H. (2019). Three decades of messenger RNA vaccine development. NANO TODAY 28, 100766. 10.1016/j.nantod.2019.100766

[B79] Walsh E. E. Frenck R. W. Falsey A. R. Kitchin N. Absalon J. Gurtman A. (2020). Safety and immunogenicity of two RNA-based covid-19 vaccine candidates. N. Engl. J. Med. 383, 2439–2450. 10.1056/NEJMoa2027906 33053279 PMC7583697

[B80] Weng Y. Li C. Yang T. Hu B. Zhang M. Guo S. (2020). The challenge and prospect of mRNA therapeutics landscape. Biotechnol. Adv. 40, 107534. 10.1016/j.biotechadv.2020.107534 32088327

[B81] Wurm T. Chen H. Hodgson T. Britton P. Brooks G. Hiscox J. A. (2001). Localization to the nucleolus is a common feature of coronavirus nucleoproteins, and the protein may disrupt host cell division. J. Virol. 75, 9345–9356. 10.1128/JVI.75.19.9345-9356.2001 11533198 PMC114503

[B82] Xia S. Duan K. Zhang Y. Zhao D. Zhang H. Xie Z. (2020). Effect of an inactivated vaccine against SARS-CoV-2 on safety and immunogenicity outcomes. JAMA 324, 951–1010. 10.1001/jama.2020.15543 32789505 PMC7426884

[B83] Zamorano Cuervo N. Grandvaux N. (2020). ACE2: evidence of role as entry receptor for SARS-CoV-2 and implications in comorbidities. eLife 9, e61390. 10.7554/eLife.61390 33164751 PMC7652413

[B84] Zhang P. Narayanan E. Liu Q. Tsybovsky Y. Boswell K. Ding S. (2021). A multiclade env–gag VLP mRNA vaccine elicits tier-2 HIV-1-neutralizing antibodies and reduces the risk of heterologous SHIV infection in macaques. Nat. Med. 27, 2234–2245. 10.1038/s41591-021-01574-5 34887575

