# Supplementary figures and images for: Single cell phototransfection of mRNAs encoding SARS-CoV2 spike and nucleocapsid into human astrocytes results in RNA dependent translation interference
Source: Front Drug Deliv. 2024 Mar 5;4:1359700. doi: 10.3389/fddev.2024.1359700 (PMC12363252; doi:10.3389/fddev.2024.1359700)

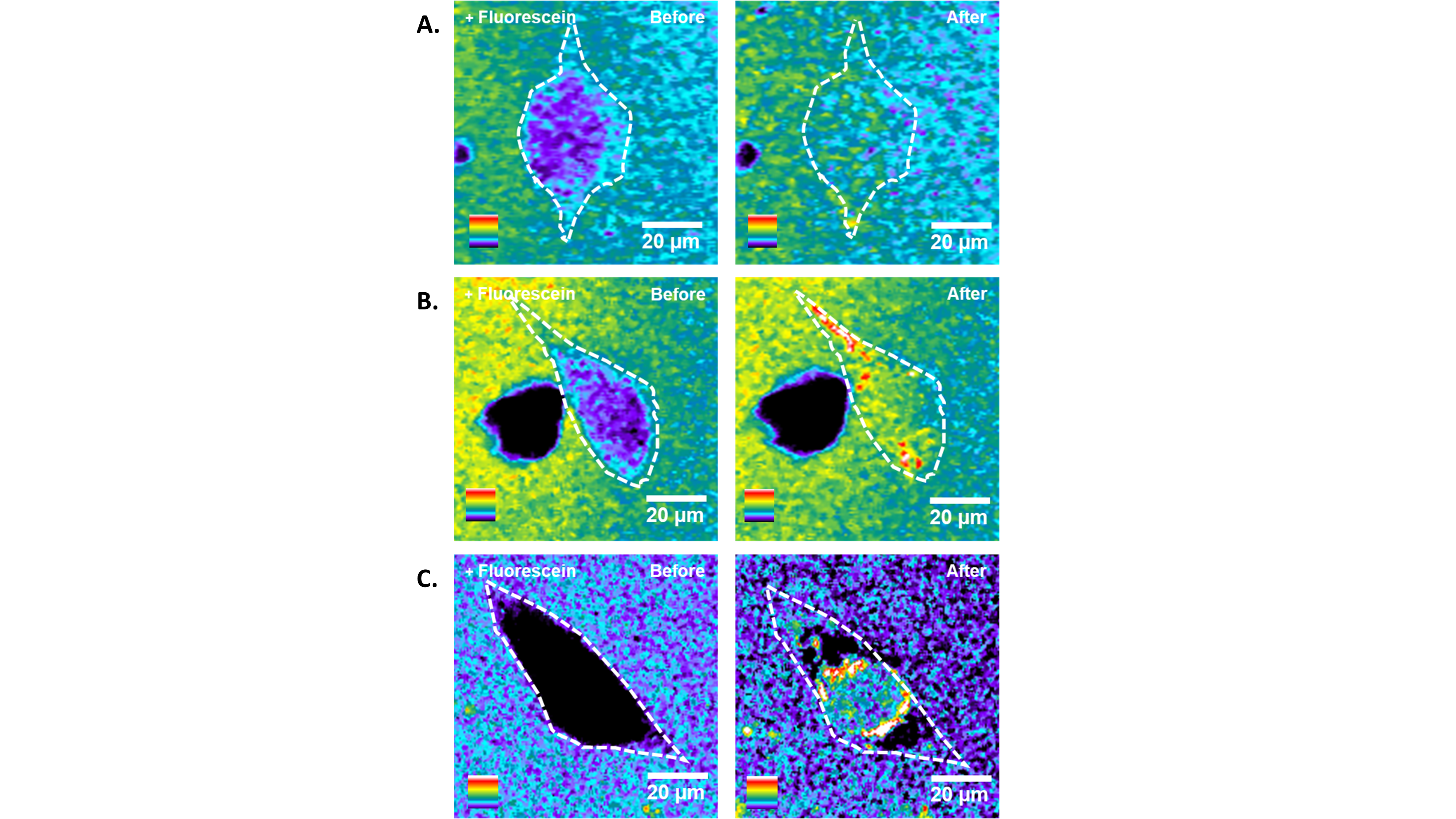

Supplement: Supplementary file 1 [file Image1.TIF]
